# Connectivity map identifies luteolin as a treatment option of ischemic stroke by inhibiting *MMP9* and activation of the PI3K/Akt signaling pathway

**DOI:** 10.1038/s12276-019-0229-z

**Published:** 2019-03-25

**Authors:** Shijian Luo, Huiqing Li, Zhihuai Mo, Junjie Lei, Lingjuan Zhu, Yanxia Huang, Ruying Fu, Chunyi Li, Yihuan Huang, Kejia Liu, Wenli Chen, Lei Zhang

**Affiliations:** 1 Department of Neurology, The Fifth Affiliated Hospital of Sun Yat-sen University, Zhuhai,, 519000 Guangdong China; 2 Department of Pharmacology, The Fifth Affiliated Hospital of Sun Yat-sen University, Zhuhai,, 519000 Guangdong China

**Keywords:** Molecular biology, Cell biology

## Abstract

This study aimed to explore potential new drugs in the treatment of ischemic stroke by Connectivity Map (CMap) and to determine the role of luteolin on ischemic stroke according to its effects on *matrix metalloproteinase-9* (*MMP9*) and PI3K/Akt signaling pathway. Based on published gene expression data, differentially expressed genes were obtained by microarray analysis. Potential compounds for ischemic stroke therapy were obtained by CMap analysis. Cytoscape and gene set enrichment analysis (GSEA) were used to discover signaling pathways connected to ischemic stroke. Cell apoptosis and viability were, respectively, evaluated by flow cytometry and an MTT (3-(4,5-dimethyl-2-thiazolyl)-2,5-diphenyl-2-H-tetrazolium bromide) assay. Quantitative real-time polymerase chain reaction (qRT-PCR) and western blot analysis were used to test the expression of MMP9 and the PI3K/Akt signaling pathway-related proteins in human brain microvascular endothelial cells (HBMECs) and tissues. Additionally, the infarct volume after middle cerebral artery occlusion (MCAO) was determined by a TTC (2,3,5-triphenyltetrazolium chloride) assay. The microarray and CMap analyses identified luteolin as a promising compound for future therapies for ischemic stroke. Cytoscape and GSEA showed that the PI3K/Akt signaling pathway was crucial in ischemic stroke. Cell experiments revealed that luteolin enhanced cell viability and downregulated apoptosis via inhibiting *MMP9* and activating the PI3K/Akt signaling pathway. Experiments performed in vivo also demonstrated that luteolin reduced the infarct volume. These results suggest that luteolin has potential in the treatment of ischemic stroke through inhibiting *MMP9* and activating PI3K/Akt signaling pathway.

## Ischemic stroke: Compound in fruit and vegetables may offer benefit

A metabolite commonly found in fruit and vegetables may provide protection against brain damage after a stroke, according to researchers in China. Lei Zhang and co-workers at Sun Yat-sen University in Guangdong employed the Connectivity Map (CMap), a tool for analyzing genome-wide data and identifying connections between diseases, genes, and potential treatments. They confirmed that the metabolite luteolin may benefit patients who have suffered ischemic stroke, which is caused by a blocked artery to the brain. Using an existing database comprising genetic details of stroke patients and controls, the team found that a major signaling pathway is inactivated and an enzyme called MMP9 is increased in stroke victims. Studies in cultured human brain cells and rats showed that luteolin can inhibit MMP9 and reactivate the signaling pathway.

## Introduction

Stroke is a major health problem, with high morbidity and mortality in developing and developed countries^1^. According to the World Health Organization, approximately 15 million people suffer from stroke per year worldwide^2^. Previous studies have revealed that the mechanisms of ischemic stroke are related to excitotoxicity, inflammation, mitochondrial dysfunction, and oxidative stress^3^. However, no approved treatments for stroke effectively interfere with these mechanisms. As the only Food and Drug Administration (FDA)-approved therapy, tissue-plasminogen activator (t-PA) is likely to increase the risk of hemorrhage and exacerbate brain damage through inflammatory reactions^3^. Other therapeutic procedures, including intravenous thrombolysis and mechanical thrombectomy^4^, also have corresponding side effects, requiring a better understanding of the basic mechanisms of stroke to develop more effective therapies.

Connectivity Map (CMap) is a systematic approach to discover functional connections between diseases, genes, and drugs through mining and comparing genome-wide transcriptional expression and changes in common gene-expression signatures in various diseases^5^. CMap covers more than 1309 compounds connected with 7000 expression profiles^6^. CMap analysis can identify drugs that affect the expression of common genes and provide a useful tool for discovering the mode of drug action, such as traditional Chinese medicine^7,8^. One study employed CMap to reveal celastrol, a pentacyclic triterpene extracted from the roots of the *Tripterygium wilfordii* plant, could be a candidate drug for the treatment of obesity by increasing leptin sensitivity to suppress food intake^9^.

Matrix metalloproteinases (MMPs) belong to the family of zinc-dependent proteolytic enzymes, which are inflammatory mediators playing an important role in physiological and pathological processes of the extracellular matrix (ECM)^10^. Until now, 23 different MMPs have been found in humans^11^. In the clinic, unbalanced MMP activity has been confirmed to be related to cardiovascular and cerebrovascular diseases^12,13^. Gelatinase B (*MMP9*) is a member of the MMP family and belongs to the group of gelatinases^11^. The expression of *MMP9* is high in human brain tissues, and *MMP9* is involved in the ischemic brain injury^14^.

The phosphatidylinositol 3-kinase (PI3K)/Akt signaling pathway involves in proliferation, inflammation, differentiation, and apoptosis^15^. Reducing apoptosis in the ischemic penumbra, where cells are potentially rescued after stroke, may be crucial^16^. In addition, some studies have shown that activation of the PI3K/Akt signaling pathway is related to the protection of ischemia/reperfusion damage in cardiomyocytes and to the upregulation of Cx43^17^. AKT regulates the activity of a variety of downstream molecules, including glycogen synthase kinase 3 beta (GSK3β), mammalian target of rapamycin (mTOR), and p70S6 kinase (P70S6K), and these proteins are combined and phosphorylated to regulate cellular metabolism^18^.

Luteolin, 3′,4′,5,7-tetrahydroxyflavone, is a member of naturally occurring compounds called flavonoids that are present widely in all kinds of fruits and vegetables^19^. It exhibits widely beneficial properties, including anti-inflammatory, anti-allergenic, anti-viral, anti-oxidant, and anti-proliferative actions^19^. In addition, it has been proven to improve rheumatoid arthritis^20^ and multiple sclerosis^21^, and to attenuate anxiety^22^. Recently, a study on the application of luteolin showed that it has anti-oxidant effects on ischemia-reperfusion injury^23^. This result indicates that luteolin may provide neuroprotection against brain damage.

In this study, microarray and CMap analyses identified luteolin as a promising compound for future therapeutics against ischemic stroke. Additionally, Cytoscape and gene set enrichment analysis (GSEA) indicated that the PI3K/AKT signaling pathway may be crucial in ischemic stroke. We studied the effect of luteolin on ischemic stroke in vitro and in vivo. The results of cell experiments revealed that luteolin was able to enhance cell viability and downregulate apoptosis by inhibiting *MMP9* and activating PI3K/AKT signaling pathway.

## Materials and methods

### Microarray analysis

The microarray profile (GSE16561) was downloaded from the Gene Expression Omnibus (GEO, https://www.ncbi.nlm.nih.gov/geo/), which contains 24 control samples and 39 stroke samples. Differentially expressed mRNAs were screened by fold change (FC) > 2, *P* < 0.05.

### Connectivity Map (CMap) analysis

To explore potential drugs targeting ischemic stroke, differentially expressed genes were divided into two parts, one for upregulation and the other for downregulation. The CMap analysis was performed through the web interface (http://www.broadinstitute.org/cmap) using version, build 02. To query the CMap, the genes were converted to probe sets in the chip type. Then, they were used as queries to search the CMap. CMap instance was measured by an enrichment score, which ranged from −1 to 1, and a permutation *P*-value.

### Gene set enrichment analysis (GSEA)

GSEA was performed based on the Kyoto Encyclopedia of Genes and Genomes (KEGG) database. The total normalized mRNA expression data were uploaded to the GSEA v3.0 software (http://software.broadinstitute.org/gsea/index.jsp). Then, KEGG pathway enrichment analysis was performed. Using the normalized enrichment scale (NES), pathways that were up- or downregulated in stroke samples compared to normal were determined. The results were performed by a GSEA enrichment plot.

### Functional network analysis

A functional network structure was constructed using the Reactome FI network Cytoscape plugin application (http://www.reactome.org/). Specifically, the differentially expressed genes were uploaded to Reactome FI, which contained information about protein–protein interactions, gene co-expression, protein domain interactions, and text mining protein interactions. The module containing the relevant gene subset was determined using a Markov Cluster Algorithm with a Pearson correlation coefficient of 0.8 and the default settings of the other parameters. The statistical significance of modules overlapping from the network analysis was measured by a hypergeometric test, where a set of populations for GO and pathway analyses were extracted from the Reactome FI and KEGG pathway databases.

### Experimental cells and animals

#### Cells

Human brain microvascular endothelial cells (HBMECs) collected from the BeNa Culture Collection (Beijing, China, http://www.bnbio.com/default.htm) were cultivated in RPMI-1640 medium with 2 mM glutamine (Sangong Biotech, Shanghai, China), 1 mM sodium pyruvate (Sangong Biotech, China), 10% fetal bovine serum (HyClone, Logan, UT, USA) and 10 mM non-essential amino acids under 5% CO_2_ at 37 °C. Cells with a density of 5 × 10^4^ cells/well in a 96-well plate were divided into 4 groups: non-oxygen and glucose deprivation/reoxygenation group (non-OGD/R): cells were non-OGD/R; oxygen and glucose deprivation/reoxygenation group (OGD/R): cells received OGD/R; DMSO (dimethyl sulfoxide) group (OGD/R + DMSO): cells received OGD/R and same volume of 1% DMSO; luteolin group (OGD/R + luteolin): cells received OGD/R and 90 μM luteolin (Shang Hai Haoran Biological Technology Co., Shanghai, China) at a purity over 90% dissolved in saline plus 1% DMSO.

#### Animals

Male Sprague Dawley (SD) rats (275–300 g, *n* = 60) were purchased from Humangen Biotech, Inc. (Shanghai, China). Rats were kept under controlled conditions. Before any experiment, they were acclimatized to the new environment for at least 7 days. Rats were divided into 6 groups for experiments: sham-vehicle group (Sham): animals received sham operation; MCAO group (MCAO): animals received MCAO; DMSO group (DMSO): animals received MCAO and same volume of 1% DMSO; MCAO with 5 mg/kg luteolin group (Luteolin-L), 10 mg/kg luteolin group (Luteolin-M), or 25 mg/kg luteolin group (Luteolin-H).

### Oxygen glucose deprivation/reoxygenation (OGD/R)

HBMECs were washed three times with phosphate buffer solution (PBS) and incubated in Earle’s balanced salt solution (116 mmol/L NaCl, 0.9 mmol/L CaCl_2_, 5.4 mmol/L KCl, 1 mmol/L NaH_2_PO_4_, 0.8 mmol/L MgSO_4_, and 10 mg/L phenol red). In a hypoxic chamber (Thermo Scientific, USA), the compact gas controller maintained the oxygen concentration at 1% by injecting a mixture of 94% nitrogen and 5% carbon dioxide gas for 2 h. After treatment with hypoxia, the cells were transferred to complete culture medium with oxygen for 24 h. Normal control cells were incubated in a regular cell culture incubator under normoxic conditions.

### Cell viability assays

Cells were plated at a density of 5 × 10^4^ cells/well in a 96-well plate. After growth for 48 h, the cells were treated with OGD/R as described above. After another 24 h, cells were used for a MTT colorimetric assay containing 4 samples: non-OGD/R group, OGD/R-treated group, OGD/R + DMSO-treated group, and OGD/R + luteolin-treated groups. Then, 110 μL of MTT (10 mg/mL) was dissolved in complete culture medium and added to each well. Plates were incubated at 37 °C for 4 h under normoxic conditions. Each well received 150 μL of DMSO and was shaken at low speed for 10 min to dissolve the dark blue solids. Then, the absorbance was read at 570 nm on a microplate reader (Thermo Scientific, USA).

### Flow cytometry

Cell apoptosis was measured by an Annexin V/PI apoptosis detection kit (Sigma, San Francisco, CA, USA) following the manufacturer’s instructions. The cell suspension was incubated at room temperature with Annexin V-FITC and PI for 10 min in the dark. For each sample, at least 1 × 10^4^ cells were analyzed using a Fluorescence Activated Cell Sorter (FACS) Calibur flow cytometer.

### Middle cerebral artery occlusion (MCAO)

All rats fasted for 1 night but were free to drink before the operation. Then, rats were anesthetized with zoletil (25 mg/kg, i.p.). The skull was exposed, and a small burr hole was made over the middle cerebral artery (MCA). A 4-0 nylon monofilament was placed underneath the right MCA rostral to the rhinal fissure, close to the major bifurcation of the right MCA, and distal to the lenticulostriate arteries. Then, the artery was lifted and the wire rotated clockwise. Both CCAs were then occluded using a microvascular clip.

### Neurological and neurobehavioral deficits

A neurological test was administered by the same examiner blinded to the experimental groups at 24 and 72 h (to determine infarct degree, *n* = 5 rats per group per time point) after MCAO following a modified scoring system according to Longa et al.^24^. The neurologic tests were scored on a five-point scale: 0 indicates no nerve function deficit; 1 indicates a mild focal neurologic deficit, and the left front paw cannot be fully extended; 2 indicates a moderate focal neurologic deficit, and the rats turn towards the left (paralysis side) in a circle when walking; 3 indicates a severe focal deficit, and the rat bodies are slumped to the left side (paralysis side) while walking; 4 indicates that the rats do not walk spontaneously and have a low level of consciousness.

### qRT-PCR

Twenty-four hours following hypoxia (1% O_2_), total RNA was extracted from HBMECs by using a RNA Simple Total RNA Extraction Kit (Invitrogen). Total RNA was reverse transcribed using a SuperScript III kit (Invitrogen), and qRT-PCR was performed using a PCR instrument (MJ, Research Opticon CFD-3200). PCR amplification was performed using specific primers, as shown in Table 1. β-Actin was used as an internal control, and the 2^−∆∆CT^ method was used to calculate the relative expression from RNA samples.

**Table 1:** Primer sequence

Primer	Sequence (5′-3′)
MMP-9 forward	5′-TGTACCGCTATGGTTACACTCG-3′
MMP-9 reverse	5′-GGCAGGGACAGTTGCTTCT-3′
Akt forward	5′-GCTGGACGATAGCTTGGA-3′
Akt reverse	5′-GATGACAGATAGCTGGTG-3′
GSK3β forward	5′-GGGACGCAGACATCGTCATC-3′
GSK3β reverse	5′-TCGTCATCGTCGAAATGGGC-3′
mTOR forward	5′-GCAGATTTGCCAACTATCTTCGG-3′
mTOR reverse	5′-CAGCGGTAAAAGTGTCCCCTG-3′
β-actin forward	5′-GGACTTCGAGCAAGAGATGG-3′
β-actin reverse	5′-AGCACTGTGTTGGCGTACAG-3′

### Western blot

The influence of luteolin on protein expression was assessed by western blot. To assess the expression of MMP9 and PI3K/Akt signaling pathway-related proteins, HBMECs and coronal sections around the infarcted area of the brain were selected for western blot. Samples were lysed in 1% sodium dodecyl sulfate buffer, pH 7.6, containing 20 mM HEPES with protease inhibitors. A BCA protein quantification kit (YEASEN Biotechnology Co., Shanghai, China) was used to measure total protein in the cell lysate. SDS-PAGE (12%) was used to separate the protein samples in equal amounts (1 μg). Then, the bands were electrotransferred onto a polyvinylidene fluoride membrane (PVDF, Millipore, MA, USA). The membrane was treated with blocking solution (0.05% Tween 20 in 20 mM TBS, 5% non-fat dry milk, pH 7.6) for 60 min at room temperature. Then, the membrane was incubated with the respective primary antibodies overnight at 4–8 °C. Antibodies to MMP9 (1:1000, ab38898), Akt (1:500, ab8805), phospho-Akt (1:500, ab38449), and GSK-3β (1:1000, ab92926) were from Abcam (Cambridge, MA, USA), and mTOR (1:2000, ab2732, Abcam, USA) was from proliferating cell nuclear antigen. Then, the cells were incubated with secondary antibodies at room temperature for 60 min. Positive immunoreactive bands were detected using an enhanced chemiluminescence kit (GE Healthcare). The intensity of protein expression was normalized to that of β-actin.

### 2,3,5-Triphenyltetrazolium chloride (TTC) staining

Infarct volume was determined by 2,3,5-triphenyltetrazolium chloride (TTC) after MCAO for 24 and 72 h (*n* = 5 per group per time point). Animals were euthanized, and the brains were collected quickly. After storage at −20 °C for 20 min, the brain tissue was cut into five coronal sections (2 mm thick), stained with 2% TTC solution at 37 °C for 20 min, and then fixed with 4% paraformaldehyde. If the tissue was dark red, it was normal tissue, and if the tissue was light gray, it was infarcted. Image analysis software was used to analyze the stained portion of TTC and calculate the infarct volume. The lesion volume was calculated as a percentage of brain edema according to the following formula: [total infarct volume − (volume of intact ipsilateral hemisphere − volume of intact contralateral hemisphere)]/contralateral hemisphere volume × 100%.

### Statistical analysis

The data were presented as the means ± standard error of the mean. Student’s *t*-test was used to perform statistical analyses. *P* < 0.05 was considered to indicate a statistically significant difference.

## Results

### Identification of differentially expressed genes and the therapeutic properties of luteolin targeting ischemic stroke

**Fig. 1:**
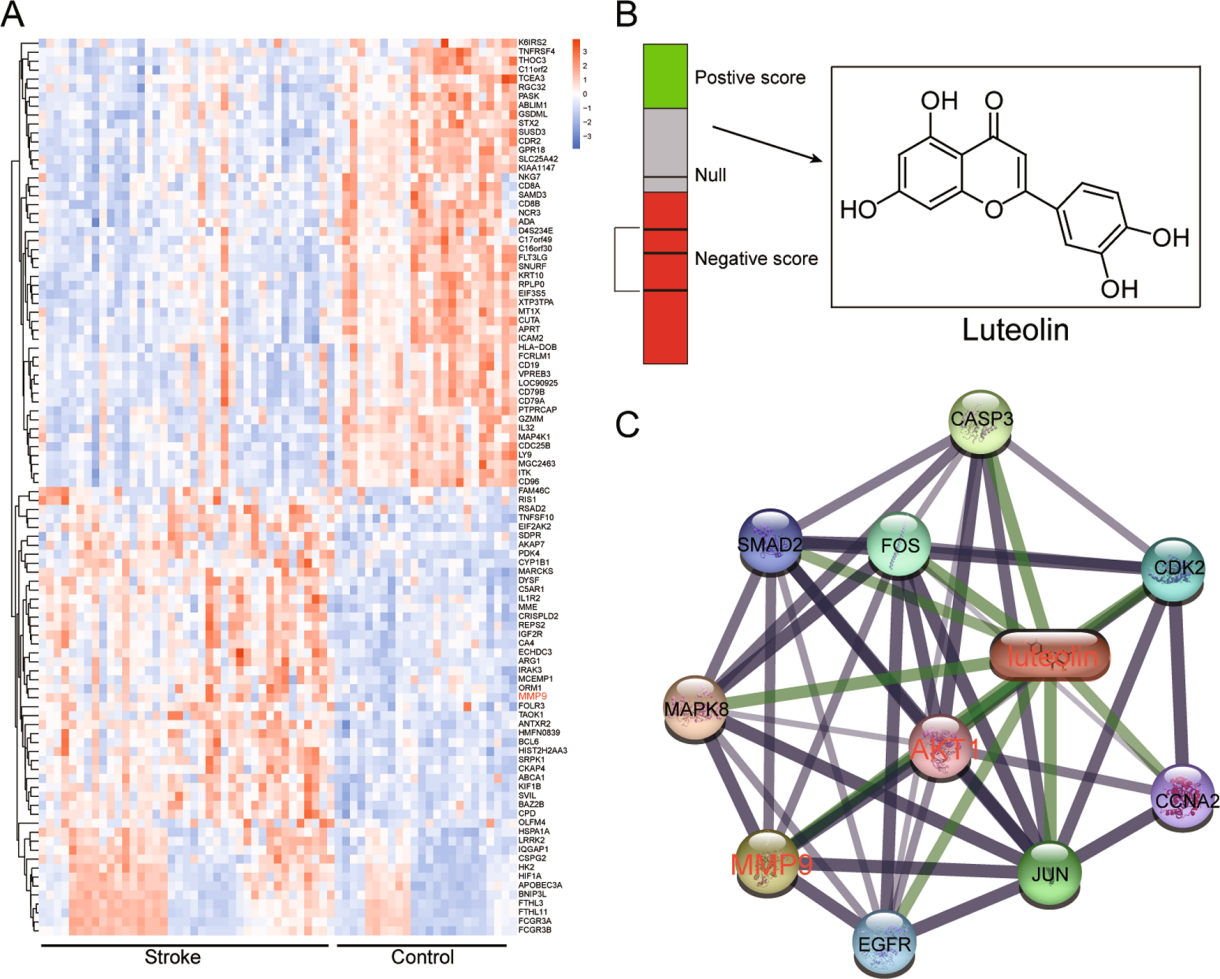
Luteolin is a potential treatment option for ischemic stroke. **a** Differentially expressed mRNAs were analyzed by microarray and presented on a heatmap. **b** Bar graph represents the connectivity score data for luteolin. The black horizontal lines represent each instance performed with the respective compound. Instances in the red area indicate negative correlation scores, and instances in the green area indicate positive ones. No correlation could be detected for instances in the gray area. The chemical structure of luteolin is also shown. **c** The target proteins of luteolin, *MMP9*, and *AKT1* were identified using STITCH

The R program was used to detect differentially expressed genes in the GSE16561 database, including 39 stroke samples and 24 control samples. In total, 493 differentially expressed genes were detected, of which 242 were upregulated and 251 downregulated. The top 50 and bottom 50 mRNAs are shown in the heatmap (Fig. 1a). To identify new treatment options for ischemic stroke, the 493 differentially expressed genes were input as queries into CMap. Disease-mimicking gene signatures generated by drugs are able to help identify pathways that represent potential therapeutic targets for corresponding diseases. Instead, a drug that induces a “reverse” signature, such as opposite changes in gene expression compared to what is observed in the disease state, could be considered as promising therapeutic agents. CMap prediction revealed that luteolin (Fig. 1b) was a potential candidate for ischemic stroke. STITCH analysis indicated that the target genes of luteolin were *MMP9* and *AKT* (Fig. 1c).

### PI3K/Akt signaling pathway is inactivated during ischemic stroke

**Fig. 2:**
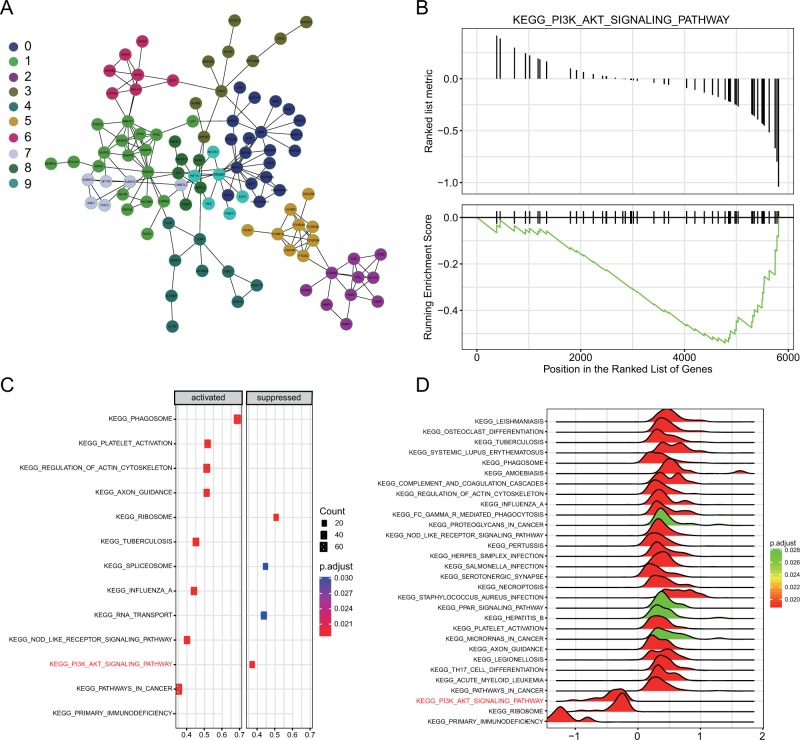
The PI3K/Akt signaling pathway is inactivated during ischemic stroke. **a** Ten functional modules from the interaction network are presented, with different colors as indicated from 0 to 9. **b** The results of GSEA indicates that the PI3K/Akt signaling pathway is inactivated during ischemic stroke. **c** The results presented as a dotplot show that the PI3K/Akt signaling pathway is downregulated during ischemic stroke. **d** The results presented as a joyplot show that the PI3K/Akt signaling pathway is downregulated during ischemic stroke

Next, we analyzed the identified signaling pathways. Using Cytoscape, we constructed a functional network structure to research the differentially expressed mRNAs, and ten dynamic functional modules were obtained. The module-related pathways showed that the PI3K/Akt signaling pathway was enriched in four modules (Fig. 2a). Using GSEA KEGG pathway analysis, we located the list of gene rankings. In the ranking and running enrichment scores, it was apparent that most genes were downregulated. The analysis showed that the PI3K/Akt signaling pathway was inactivated (Fig. 2b). As shown in Fig. 2c, d, dotplot and joyplot show the rankings of signaling pathways that were upregulated or downregulated. The PI3K/Akt signaling pathway was downregulated by dotplot and joyplot. Therefore, in the following studies, these results prompted us to focus on the PI3K/Akt signaling pathway.

### Effect of luteolin on cell viability and apoptosis

**Fig. 3:**
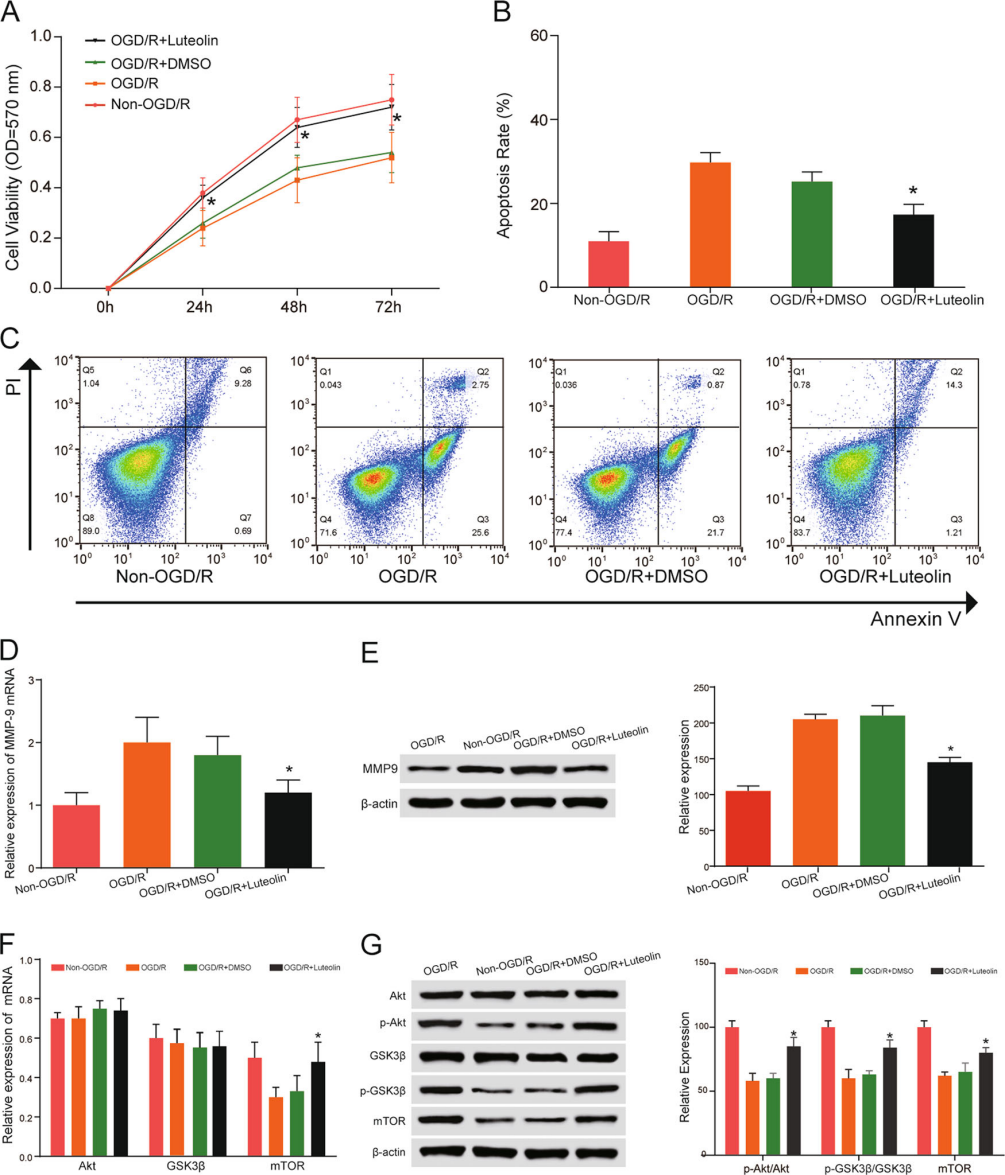
Effect of luteolin on HBMECs. **a** The proliferation of HBMECs was assessed in the MTT experiment. The results showed that OGD/R can inhibit their proliferation, while luteolin treatment can promote their proliferation. **b**, **c** The apoptosis of HBMECs was assessed by flow cytometry, and the results showed that OGD/R can enhance apoptosis, while apoptosis was downregulated upon luteolin treatment. *P < 0.05, compared with the same time-point in the OGD/R group. **d** The mRNA of *MMP9* was detected by qRT-PCR, and the results showed that luteolin enhances the mRNA of *MMP9*. *P < 0.05, compared with the OGD/R group. **e** The protein levels of MMP9 could be suppressed by luteolin. *P < 0.05, compared with the same time-point in the OGD/R group. **f** The expression levels of PI3K/Akt signaling pathway mRNAs were upregulated and activated by luteolin. *P < 0.05, compared with the same time-point in the OGD/R group. **g** The protein levels of the PI3K/Akt signaling pathway proteins were upregulated and activated by luteolin. *P < 0.05, compared with the same time-point in the OGD/R group

We assessed the effects of luteolin on the viability and apoptosis of HBMECs. The viability of HBMECs was determined by MTT assay. As shown in Fig. 3a, the viability of HBMECs was enhanced after exposure to 90 μM luteolin from 24 h (OGD/R + luteolin group) to 72 h. In addition, apoptosis of HBMECs was evaluated following the exposure of cells to 90 μM of luteolin (OGD/R + luteolin group). Flow cytometry confirmed that luteolin reduced apoptosis of HBMECs (OGD/R + luteolin group). As shown in Fig. 3b, c, the ratio of apoptotic cells was proportional to the treatment time of luteolin. The results showed that luteolin enhanced cell viability and downregulated apoptosis.

### Luteolin potentially reduces mRNA and protein expression of *matrix metalloproteinase-9* (*MMP9*)

**Fig. 4:**
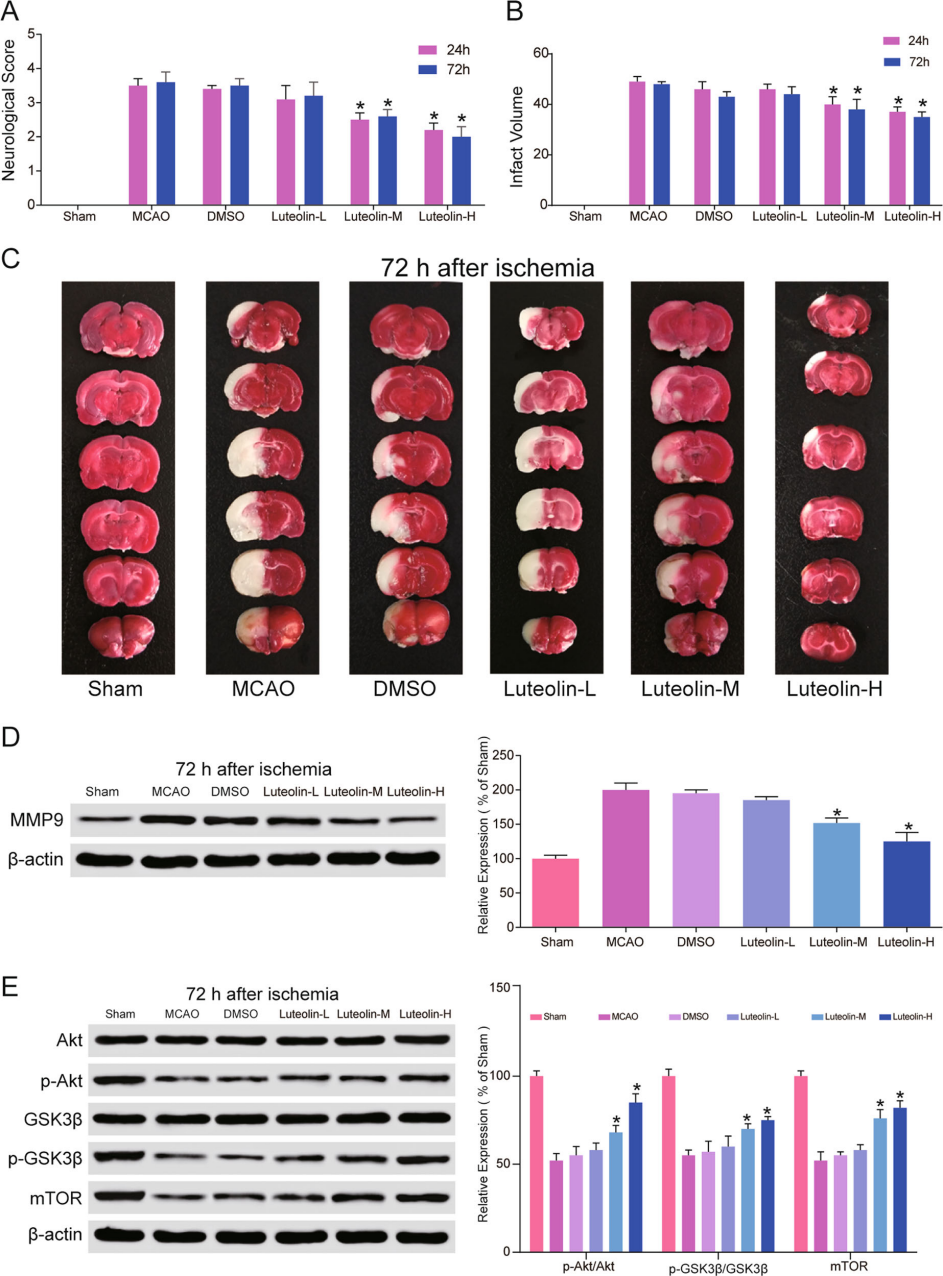
Effect of luteolin on ischemic rats’ brains. **a** Compared with the DMSO group, the neurologic deficit scores were significantly decreased in the luteolin-M group and luteolin-H group at 24 and 72 h, but no significant differences were found in the luteolin-L group. *P < 0.05, compared with the same time-point in the DMSO group. **b**, **c** Compared with the DMSO group, the infarct volume was significantly reduced in the luteolin-M group and luteolin-H group at 24 and 72 h, but no significant differences were found in the luteolin-L group. *P < 0.05, compared with the same time-point in the DMSO group. **d** The expression levels of MMP9 were suppressed by luteolin in coronal sections around the infarcted area of the brain. *P < 0.05, compared with the same time-point in the DMSO group. **e** The expression levels of PI3K/Akt signaling pathway-associated proteins were upregulated and activated by luteolin in coronal sections around the infarcted area of the brain. *P < 0.05, compared with the same time-point in the DMSO group

MMPs play a vital role in the degradation of the ECM, and one MMP was a target protein of luteolin predicted to be upregulated by microarray. We determined the mRNA and protein expression of *MMP9* under the influence of luteolin in HBMECs. As shown in Fig. 3d, e, *MMP9* mRNA and protein levels were decreased by luteolin in HBMECs. We also detected the expression of *MMP9* in coronal sections around the infarcted area of the brain. The results were similar (Fig. 4d). With the 10 mg/kg (luteolin-M) and 25 mg/kg (luteolin-H) groups, the results showed more obvious suppression than the 5 mg/kg (luteolin-L) group. Therefore, luteolin inhibits the expression of MMP9 in both HBMECs and tissues.

### Luteolin activates the PI3K/Akt signaling pathway

It has been reported that activation of the PI3K/Akt signaling pathway is important for neuroprotection against ischemia-induced apoptosis^25^. The pathway analysis showed that the PI3K/Akt signaling pathway was important in ischemic stroke. Therefore, we detected the mRNA and protein expression of PI3K/Akt signaling pathway members by qRT-PCR and western blot in HBMECs, which are shown in Fig. 3f, g. Compared to the OGD/R group, GSK3β and mTOR mRNA were increased in the OGD/R + luteolin groups. However, there were no apparent differences in Akt mRNA between each group (all *P* > 0.05). The expression of p-Akt, GSK3β, and mTOR was suppressed in HBMECs. After treatment with luteolin, the levels of p-Akt/Akt, p-GSK3β/GSK3β, and mTOR were clearly elevated. The same results were found in tissues (Fig. 4e). Protein expression was clearly increased in the luteolin-M and luteolin-H groups compared with the DMSO group. These results indicate that the PI3K/Akt signaling pathway is activated by luteolin both in HBMECs and tissues.

### Luteolin reduces infarct

Figure 4a shows the scoring of neurological behavior at 24 and 72 h after MCAO. Rats in the Sham-vehicle group had a score of zero, while those in the MCAO group had a high score. After treatment with high concentrations of luteolin (25 mg/kg), the neurological score decreased significantly compared with the MCAO group. Infarct volume was measured by TTC at different time points after MCAO. At 24 and 72 h after surgery, the infarct area for each group is shown in Fig. 4b. After TTC staining, dark red indicates normal tissue, and light gray indicates infarct area. In the Sham-vehicle group, no infarction was observed. In the MCAO group, the area of infarction was easily observed. The effects of treatment with different concentrations of luteolin showed different results after 24 and 72 h. There was an efficacious reduction in infarct area in the luteolin-M and luteolin-H groups compared with the MCAO group. Meanwhile, in luteolin-L group, the effect was not apparent (Fig. 4c). On the basis of these results, we conclude that daily treatment with the middle dose (10 mg/kg) and high dose (25 mg/kg) of luteolin after stroke would have better therapeutic effects.

## Discussion

In this study, through bioinformatics analysis, we found that *MMP9* is highly expressed and the PI3K/Akt signaling pathway is inactive in stroke samples. CMap analysis via website prediction identified luteolin as a promising compound for ischemic stroke therapy. Cell experiments revealed that luteolin enhanced cell viability and downregulated apoptosis by inhibiting the expression of *MMP9* and activating the PI3K/Akt signaling pathway. In vivo experiments also displayed that luteolin could reduce the infarct. This result may explain the clinical utility of luteolin to a certain extent and will contribute to subsequent research.

CMap is used to identify new potential targets by establishing relationships between gene expression patterns and disease. Based on CMap, the mechanisms of Xuesaitong injection (XST), a Chinese medicine of *Panax notoginseng* roots that has been used for the prevention and treatment of stroke, were found^6^. In our study, CMap identified the therapeutic properties of luteolin for ischemic stroke. Recent studies reported that apigenin has protective effects on both neuronal and glial cells during stroke^26,27^. Furthermore, Withaferin A and Carvacrol were also found to protect against cerebral infarction^1,28^.

Experimental data have shown that MMPs play an important role in cell migration and degradation of the ECM^29^. *MMP3* has been associated with vascular risk and can predict the risk of ischemic stroke and myocardial infarction^30^. In the study of Zhang et al., downregulation of *MMP2* and *MMP9* inhibited cell migration^1^. Chen et al. reported a similar result in a rodent model of cerebral ischemia^31^. In our study, based on microarray analysis and in vitro experiments, *MMP9* was upregulated in stroke samples. In addition, the PI3K/Akt signaling pathway was suppressed in ischemic stroke. In the study of Xu et al., they found that the PI3K/Akt signaling pathway mediated neuroprotective effects on cerebral ischemia/reperfusion injury^32^. Previous studies also revealed that activating the PI3K/Akt signaling pathway could attenuate ischemia/reperfusion (I/R) injury and protect against neurogenesis^33,34^. Activation of PI3K/Akt resulted in the inhibition of cerebral cell apoptosis^35^.

Luteolin has been reported to have therapeutic effects on many diseases, such as multiple sclerosis (MS)^36^ and Alzheimer’s disease (AD)^37^. In addition, luteolin inhibits the I/R-induced myocardial infarct size in vivo^38^ and inhibits proliferation and induces apoptosis of human placental choriocarcinoma cells by blocking the PI3K/Akt signaling pathway^15^. In our study, luteolin would be a treatment option for ischemic stroke. Cell experiments demonstrated that luteolin enhanced cell viability and down-regulated apoptosis. After treatment with luteolin, the expression level of *MMP9* was decreased, and proteins in the PI3K/Akt signaling pathway were increased. In vivo experiments also showed that luteolin could clearly reduce the infarct volume. Similar results were also found in the study of Yang et al., where luteolin had protective effects against ischemic stroke through the reduction of apoptosis and oxidative stress; downregulation of *MDA* and *Bax*; and upregulation of *SOD1*, *CAT*, *Bcl-2*, and *Claudin-5*^39^.

In conclusion, *MMP9* was upregulated, and the PI3K/AKT signaling pathway was inactive during ischemic stroke. Luteolin was able to enhance cell viability and downregulate apoptosis by downregulating the expression levels of *MMP9* and activating the PI3K/Akt signaling pathway. In vivo experiments showed that luteolin could reduce the infarct volume. These results suggest that luteolin may be a potential therapeutic drug for ischemic stroke. Further studies are needed to verify our findings, to examine the mechanisms of luteolin for ischemic stroke, and to determine whether luteolin could improve clinical outcomes.
